# A Simple Stomatoscope

**Published:** 1883-04

**Authors:** C. M. Wright

**Affiliations:** Cincinnati


*Communications—Continued from Page* 162.

### A Simple Stomatoscope.

BY C. M. WRIGHT, CINCINNATI.

Sometimes, nay often, in large cities like London, Cincinnati and Riley, O., the dentist finds it necessary to illuminate the oral cavity by artificial appliances, and many have given thought to this matter. In London some dental offices are brilliantly lighted by calcium lights. Glass globes filled with distilled water—the water in some cases being slightly tinted—are arranged as reflectors and used by many operators. The majority of dentists have simply open gas jets flaring and fizzing in front of their operating chairs, employing this imperfect light only occasionally. For a few years past a modification of the Tobold laryngeal illuminator has been employed in Germany, known as the "Dr. Grohnwald's Stomatoscope," and now we have in the United States almost an exact imitation of this, offered for sale as the "Beseler Stomatoscope." That these lamps are improvements on the day light often furnished in large cities, and on dark days even in the country, there can be no doubt; but, that *every* dentist in every town, *should* find a clinical dental lamp, or stomatoscope, of frequent advantage even on bright days, is an idea that has not perhaps fully penetrated the dentist's brain. The proper illumination of the oral cavity is as important a matter to the dentist as the illumination of the larynx or pharynx is to the throat specialist; or the illumination of the *object* is to the accomplished microscopist; and as sunlight, reflected from a white cloud, or directed by a system of reflecting lenses or mirrors into the oral cavity is not always available, some form of artificial illumination should be at hand in every well regulated dental office, so that strong light could be controlled and directed into any part of the mouth, and even half or three-quarters of the distance up or down a pulp canal in a posterior tooth.

Any one who has studied the subject and experimented practically knows this, and would not be without his clinical lamp, and his skill in the use of a reflecting mouth mirror and lenses.

Having had considerable experience in the matter and having employed nearly all the methods now known for artificial illumination, from the simple tube with a bull's eye condensor in one end, arranged to fit over a candle or lamp chimney, such as Morell Mackensie, of London, has devised, to the electric light at the handle of a mouth mirror, and also the Grohnwald stomatoscope, I beg leave to offer a description of the most convenient and simple lamp that I have ever employed. The Beseler stomatoscope, though of great value, is a clumsy article of furniture, and must be adjusted by the assistant, or by raising or lowering by lifting, and fastening with a screw, etc., and the reflected light from the concave mirror is no better than direct light condensed by a lens. Then the lamp is hot, for a great deal of the *light* is wasted. My simple lamp is very much cheaper, less complicated, and can be used for ordinary purposes. Well, now, I fitted a rod into the *hand* of the *How's adjustable bracket*, after removing the table and, taking the lamp and reservoir of the *American student lamp*, in which head light oil is burned, from the standard, slipped it on the rod in the bracket. This looks neat, and has all the appearance of an original design, *i. e.*, it looks as though the bracket had been invented to carry in its miniature hand a student lamp. I employ this bracket for no other purpose, having my clinical lamp always ready. When lighted this sheds a mild, soft, steady light *above*, at the *side*, or wherever you may want light and wish to avoid the annoying shadows of the hand. When it is desirable to concentrate a strong light, as a laryngeal lamp, or stomatoscope, place a bull's eye, or other condensor, between the flame and the object. To have this handy, removable and simple, I cut off the door of an old-fashioned "dark lantern," in which a bull's eye is set, and this forms the whole of the apparatus. I simply lay this door with its bull's eye lens under the ring that holds the white porcelain shade of the lamp, and direct the rays which are condensed by it into the mouth. It forms a circle that can be kept out of the eyes of the patient, and concentrates a light strong enough for our purpose—as strong indeed as the light from the more elaborate stomatoscope. This can all be arranged with one hand while standing at the chair, and a strong condensed light, or a mild soft light can be had at pleasure, or the light can be reduced and the lamp pushed out of the way while the left hand is holding a napkin in the patient's mouth. Then by having the bracket fastened to the left of the chair as I have mine, the lamp can be pushed back of the patient, and with a laryngeal concave mirror fastened to the forehead or on a spectacle frame the operator can illuminate the mouth as brilliantly as need be for operations on the tonsils, etc.

The nickel-plated student lamp costs about $4, and it can be removed from the bracket and slipped back on its standard and employed as a reading lamp anywhere. The bracket serves admirably as a lamp holder on account of its great range and ease of motion, and if necessary one can make one bracket answer for a lamp carrier and a table carrier, if he will take the trouble to change and will do without the table while using the lamp. In short, for economy and simplicity and effectiveness it is quite perfect, and I should not be ashamed to have it christened "Wright's Clinical Dental Lamp," although Dr. How invented the bracket, some other good fellow invented the student lamp, and still some other, but more ancient person, before Pickwick's time, invented the bull's eye lantern. I only invented the little piece of rod about four inches long that is stuck into the hand, and *combined* the products of these *other* inventive geniuses into the "clinical dental lamp." Try it.

